# MicroRNA-320a sensitizes tamoxifen-resistant breast cancer cells to tamoxifen by targeting ARPP-19 and ERRγ*

**DOI:** 10.1038/srep08735

**Published:** 2015-03-04

**Authors:** Mingrong Lü, Keshuo Ding, Guofeng Zhang, Mianmian Yin, Guidong Yao, Hui Tian, Jie Lian, Lin Liu, Meng Liang, Tao Zhu, Fei Sun

**Affiliations:** 1 Institute of Immunology and CAS Key Laboratory of Innate Immunity and Chronic Disease, Innovation Center for Cell Biology, School of Life Sciences and Medical Center, University of Science and Technology of China, Hefei, Anhui 230027, People's Republic of China; 2 Hefei National Laboratory for Physical Sciences at Microscale, Hefei, Anhui 230027, People's Republic of China; 3 Department of Pathology, Anhui Medical University, Hefei, Anhui, People's Republic of China; 4 Department of General Surgery, Tongji Hospital, School of Medicine, Tongji University, Shanghai 200065, People's Republic of China; 5 Reproductive Medical Center, The First Affiliated Hospital of Zhengzhou University, Zhengzhou, Henan 450052, People's Republic of China

## Abstract

Tamoxifen represents a major adjuvant therapy to those patients with estrogen receptor-alpha positive breast cancer. However, tamoxifen resistance occurs quite often, either *de novo* or acquired during treatment. To investigate the role of miR-320a in the development of resistance to tamoxifen, we established tamoxifen-resistant (TamR) models by continually exposing MCF-7 or T47D breast cancer cells to tamoxifen, and identified microRNA(miRNA)-320a as a down-regulated miRNA in tamoxifen resistant cells. Re-expression of miR-320a was sufficient to sensitize TamR cells to tamoxifen by targeting cAMP-regulated phosphoprotein (ARPP-19) and estrogen-related receptor gamma (ERRγ) as well as their downstream effectors, c-Myc and Cyclin D1. Furthermore, progesterone (P_4_) promoted the expression of miR-320a by repressing c-Myc expression, while estrogen (E_2_) exerted the opposite effect. These results suggest the potential therapeutic approach for tamoxifen-resistant breast cancer by restorating miR-320a expression or depleting ARPP-19/ERRγ expression.

Breast cancer is one of the most commonly detected cancers in women and a leading cause of cancer deaths worldwide^1^. Approximately 70% of breast cancer patients overexpress the nuclear receptors, including estrogen receptor-alpha (ERα)/progesterone receptor (PR), making it an exceptional candidate for endocrine therapy. Tamoxifen (TAM), as a selective estrogen-receptor modulator (SERM) which represses ERα activity by competitively inhibiting the interaction of estrogen with ERα, is commonly administered as the first-line adjuvant treatment of ER-positive (ER+) patients. However, up to 50% of ER+ patients with metastatic diseases do not respond to tamoxifen treatment and many initial responders relapse eventually^2^,^3^. A number of mechanisms have been proposed to explain anti-estrogen resistance in ER+ breast cancer. Among those, the overexpression of estrogen-targeted cell cycle regulatory molecules c-Myc/Cyclin D1^4^,^5^,^6^,and estrogen-related receptor-gamma (ERRγ) have been associated with tamoxifen resistance^2^. The knockdown of ERRγ in SUM44/LCC-TamR cells restores tamoxifen sensitivity, and overexpression of ERRγ blocks the growth-inhibitory effects of tamoxifen in SUM44 and MDA-MB-134 VI lobular breast cancer cells^2^. Recently, microRNAs (miRNAs) have also indicated a critical role in mediating tamoxifen resistance by regulating their target genes^7^.

The miRNAs are a class of small, non-coding RNAs that post-transcriptionally control the translation and stability of mRNAs^8^,^9^. Dysregulated miRNA expression is frequently associated with the development of many types of human tumors. Almost half of the known human miRNAs are located in cancer-associated genomic regions or fragile sites^10^. The involvement of miRNAs in tamoxifen resistance has been previously described. For example, miR-221/222 was able to confer tamoxifen resistance^11^, while re-expression of miRNA-375, let-7, or miR-342 induced tamoxifen sensitivity by down-regulating their target genes^3^,^9^,^12^. Mmu-miR-320, one of the most significantly down-regulated miRNAs in TGF-β1-treated mouse ovarian granulosa cells (GCs)^13^, inhibited E_2_ synthesis and GC proliferation, but promoted progesterone production through targeting E2F1 and SF-1^14^. In addition, ARPP-19 (cAMP-regulated phosphoprotein), a target of miR-320a, is present at high levels in human malignant cell lines and in the embryos^15^,^16^,^17^. These results indicate that miR-320a may play a role in steroid-related disorders. In this study, the roles of miR-320a in the regulation of the tamoxifen sensitivity of ER+ breast cancer cells were investigated by identifying its target genes and downstream regulators.

## Results

### MiR-320a directly targets ARPP-19/ERRγ in breast cancer cell lines

**Figure 1:**
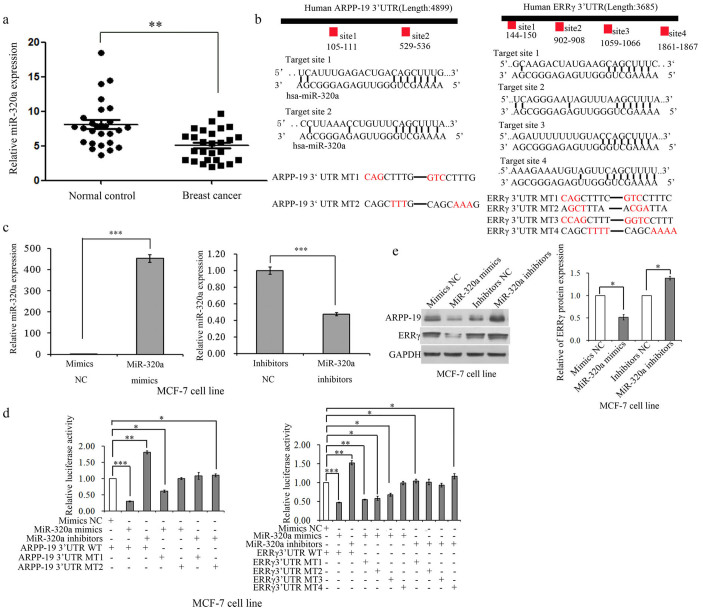
miR-320a directly targets ARPP-19 and ERRγ in MCF-7 cell line. (a). miR-320a expression levels were examined in 58 fresh tissues including 31 breast cancer and 27 normal breast samples from patients using real-time PCR. (b). Putative-binding sites for human (hsa) miR-320a were predicted in the 3′UTR of ARPP-19 and ERRγ mRNA and mutation sites. (c). Real-time PCR analysis of miR-320a expression in MCF-7 cells when transfected with miR-320a mimics or inhibitors. (d). Luciferase activity of luciferase gene fusing with wild-type or mutant 3′UTRs of ARPP-19 or ERRγ were measured after co-transfection with miR-320a mimics or inhibitors in MCF-7 cell line. The luciferase activity was normalized with firefly luciferase activity. (e). Western blot analysis of the expression levels of ARPP-19 and ERRγ in MCF-7 cells transfected with miR-320a mimics or inhibitors and the densitometric analysis of ERRγ protein levels. Results are presented as an average of at least three replicates.*P < 0.05, **P < 0.01, ***P < 0.001.

In our previous study, we have demonstrated the role of miR-320 in granulose cells^14^. For further study of this microRNA, we first evaluated the expression of miR-320a in human tissues taken from patients and revealed that miR-320a expression levels were significantly lower in breast tumor tissues compared with normal breast tissues (Fig. 1a), which indicated that miR-320a may play an anti-tumor role in breast cancer cells. In addition, we did not identify the relationship between the expression of miR-320a and of ER/PR/HER2 in 31 breast cancer tissues (Supplementary Table 1). However, whether miR-320a expression is correlated with breast cancer subtypes is needed to be examined in more extensive patient cohorts. Using multiple databases, including TargetScan, PicTar, and miRanda, two conserved miR-320a target sites in the ARPP-19 3′ UTR and four target sites in the ERRγ 3′ UTR (Fig. 1b) were predicted, respectively. To evaluate the efficiency of miR-320a mimics and inhibitors, we performed real time PCR assay. Fig. 1c and Fig. S1a showed that miR-320a increased significantly after being transfected with mimics and decreased significantly after being transfected with inhibitors. We next examined whether ARPP-19 and ERRγ were the direct targets of miR-320a. As shown in Fig. 1d and Fig. S1d, the luciferase reporter activity was significantly suppressed by miR-320a mimics when transfected with the reporter plasmids containing 3'UTR of either ARPP-19 or ERRγ, whereas miR-320a inhibitors increased the luciferase activity in MCF-7 and T47D cell lines. Mutations of the predicted target sequences of the 3′UTR of ARPP-19 and ERRγ (Fig. 1b), can partially (Mutation-1 for ARPP-19 and Mutation-1, 2, 3 for ERRγ), or almost completely (Mutation-2 for ARPP-19 and Mutation-4 for ERRγ), rescue the suppressive effect of miR-320a (Fig. 1d). In addition, the miR-320a inhibitors incresed the luciferase activity when transfected with the mutation plasmids compared with co-transfected with the mimic negative control (mimic NC) and the wild-type (WT) plasmids (Fig. 1d and Fig. S1d). Concordantly, overexpression or depletion of miR-320a significantly decreased or increased ARPP-19 and ERRγ at protein levels in MCF-7 cells (Fig. 1e) and T47D cells (Fig. S1e) as well as ARPP-19 mRNA (left panel of Fig. S1b/c). However, miR-320a did not affect ERRγ mRNA levels (right panel of Fig. S1b/c), indicating that ERRγ was regulated by miR-320a at translation level.

### Knockdown of miR-320a reduced sensitivity of tamoxifen in ER+ breast cancer cell lines

**Figure 2:**
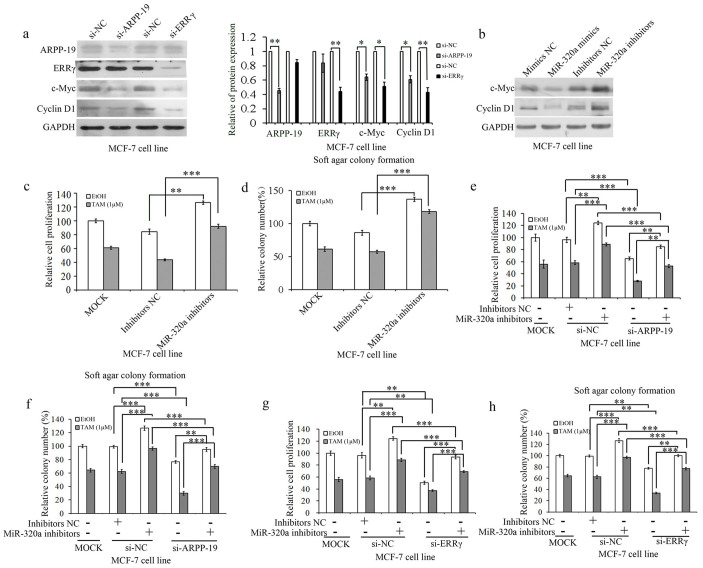
Knockdown of miR-320a reduced sensitivity of tamoxifen in MCF-7 cells. (a). Western blot analysis of ARPP-19, ERRγ, c-Myc, and Cyclin D1 expression levels in MCF-7 cells transfected with si-ARPP-19 and si-ERRγ and the corresponding densitometric analysis. (b). Western blot analysis of c-Myc and Cyclin D1 expression levels in MCF-7 cells transfected with miR-320a mimics/inhibitors. c and d. Cell viability (c) and soft agar colony formation (d) analysis of MCF-7 cells transfected with miR-320a inhibitors. e and f. Cell viability (e) and soft agar colony formation (f) analysis of MCF-7 cells transfected with si-ARPP-19 and miR-320a inhibitors and co-transfected with si-ARPP-19 and miR-320a inhibitors on exposure to tamoxifen or not. f and g. Cell viability (f) and soft agar colony formation (g) analysis of MCF-7 cells transfected with si-ERRγ and miR-320a inhibitors and co-transfected with si-ERRγ and miR-320a inhibitors on exposure to tamoxifen or not. Results are presented as an average of at least three replicates. *P < 0.05, **P < 0.01, ***P < 0.001.

To investigate the downstream signaling of ARPP-19 and ERRγ, we studied some genes that were associated with cell growth or cell migration and invasion. As shown in Fig. 2a and Fig. S2a, the silencing of ARPP-19 and ERRγ resulted in a significant decrease in the levels of c-Myc and Cyclin D1 proteins in both MCF-7 cells and T47D cells. These results indicate that c-Myc and Cyclin D1 were regulated by ARPP-19 and ERRγ and may be involved in miR-320a regulation. Concordantly, the expression of c-Myc and Cyclin D1 was reduced by miR-320a overexpression and, conversely, the miR-320a depletion led to elevated levels of these proteins (Fig. 2b and Fig. S2b). As previously reported, c-Myc and Cyclin D1 were strongly associated with the development of tamoxifen resistance^5^,^18^, we wanted to know whether miR-320a and its targets could also be involved in the regulation of tamoxifen sensitivity in ER+ breast cancer cells. MCF-7 and T47D cells were transfected with miR-320a inhibitors. As shown in the Fig. 2c/2d and Fig. S2c/S2d, miR-320a inhibitors increased the cell viability and colony formation of MCF-7/T47D cells without tamoxifen treatment compared with their negative controls; and on exposure to tamoxifen, miR-320a inhibitors rescued the repressor induced by tamoxifen. This result indicates that miR-320a inhibitors reduced the sensitivity of ER+ breast cancer cells to tamoxifen. In addition, knockdown of ARPP-19 and ERRγ inhibited MCF-7 and T47D cells' growth whether exposed to tamoxifen or not as determined by cell viability analysis (Fig. 2e/2g and Fig. S2e/S2g) and soft agar colony formation assay (Fig. 2f/2h and Fig. S2f/S2h). Meanwhile, miR-320a inhibitors enhanced the resistance of MCF-7 and T47D cells to tamoxifen, whereas the knockdown of ARPP-19 and ERRγ in miR-320a inhibitors-transfected MCF-7 or T47D cells showed restored sensitivity to tamoxifen, both in cell viability analysis and soft agar colony formation assay (Fig. 2e–2h, Fig. S2e–S2h and Supplementary Table 2).

### Tamoxifen resistant cell lines showed a low level of miR-320a and a high level of c-Myc, Cyclin D1, ARPP-19 and ERRγ

**Figure 3:**
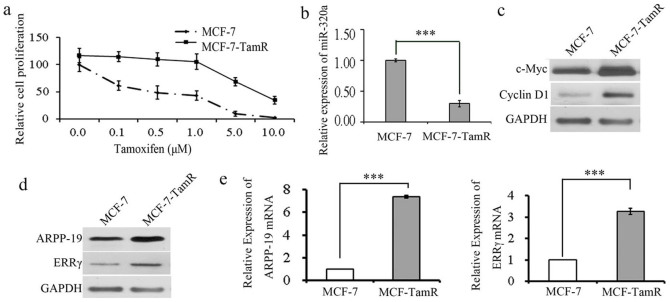
Tamoxifen resistant cell lines had a low level of miR-320a and a high level of c-Myc, Cyclin D1, ARPP-19, and ERRγ. (a). Cell viability analysis of MCF-7-TamR cells as well as its parental cells treated with different doses of tamoxifen. (b). Real-time PCR analysis of miR-320a expression in TamR cells compared with parental cells. (c) and (d). Western blot analysis of the expression of c-Myc, Cyclin D1, ARPP-19 and ERRγ in MCF-7-TamR cells as well as parental cells. (e). Real-time PCR analysis of ARPP-19 and ERRγ mRNA expression in TamR cells compared with parental cells. Results are presented as an average of at least three replicates. ***P < 0.001.

**Figure 4:**
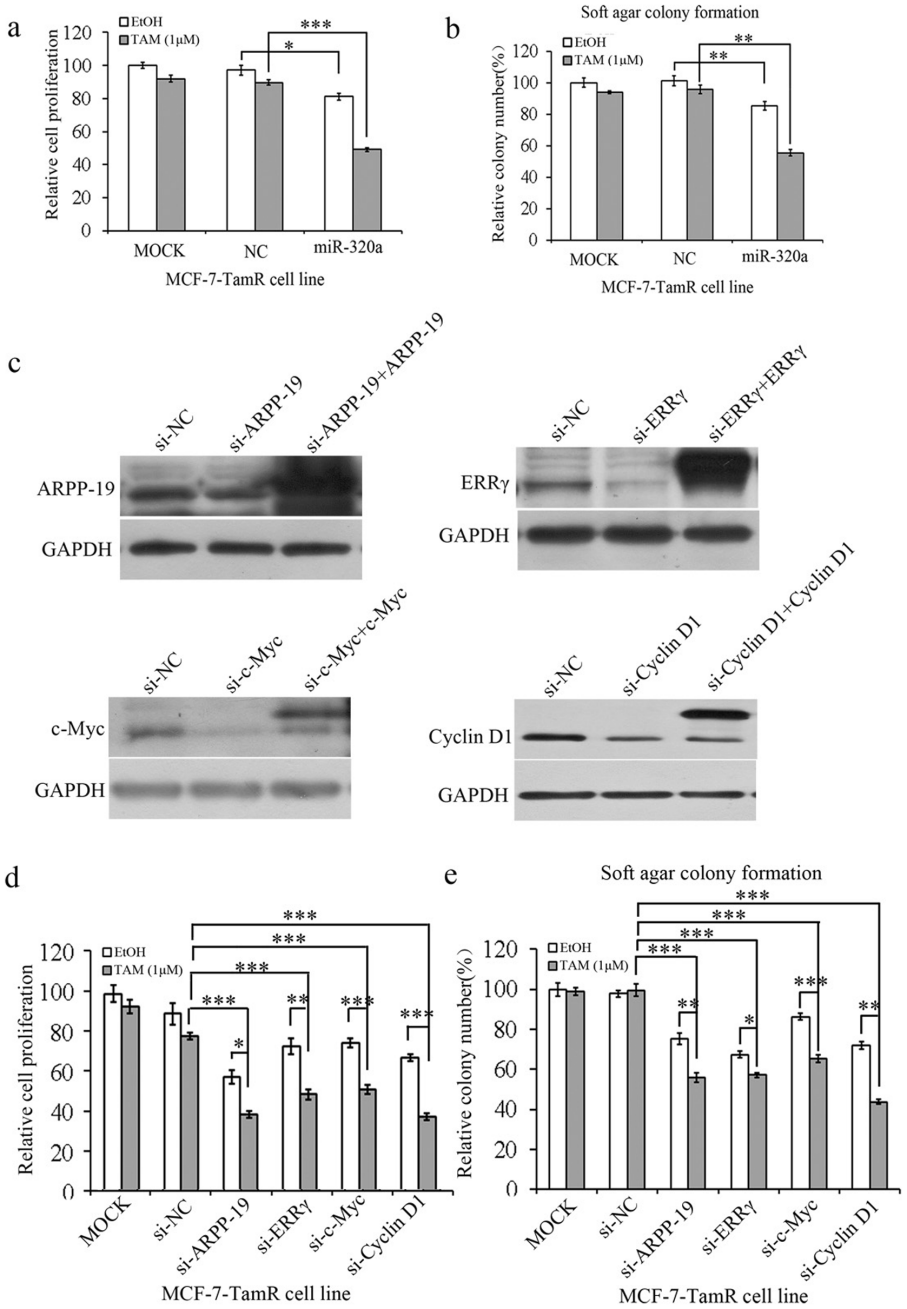
Re-expression of miR-320a or knock-down of its targets in TamR cells sensitizes these cells to tamoxifen. (a) and (b). Cell viability (a) and soft agar colony formation (b) analysis of TamR cells transfected with miR-320a mimics. (c). The expression of ARPP-19, ERRγ, c-Myc, and Cyclin D1 was examined by Western blot after transfected with indicated siRNA or siRNA and plasmids for 48 h. ARPP-19, c-Myc, and Cyclin D1 were labeled with FLAG tag and ERRγ was labeled with GFP tag. d and e. Cell viability (d) and soft agar colony formation (e) analysis of TamR cells transfected with si-ARPP-19, -ERRγ, -c-Myc, and -Cyclin D1. Results are shown as the average and s.d. of at least three biological and two technical replicates each,*P < 0.05, **P < 0.01, ***P < 0.001.

We next established tamoxifen resistant breast cancer cell lines MCF-7 and T47D (designated as MCF-7/T47D-TamR cells) by continuously exposing them to 1 μM tamoxifen for at least 12 months to study the role of miR-320a. The tamoxifen resistance was validated by testing the viability of these cell lines using CCK8 (Fig. 3a and Fig. S3a). The expression of miR-320a was examined in MCF-7-TamR and T47D-TamR cells. As shown in Fig. 3b and Fig. S3b, the expression of miR-320a was significantly reduced in TamR cells compared with their parental cells. In accordance with the reports^12^ that the expression of let-7i was reduced in MCF-7-TamR cells compared with their parental cells and that it was not changed inT47D-TamR cells compared with their parental cells (Fig. S4a/b). Over expression of let-7i increased the sensitivity of MCF-7-TamR cells to tamoxifen (Fig. 4c) and knockdown of let-7i reduced the sensitive of MCF-7 cells to tamoxifen (Fig. S4d). Accordingly, a dramatic increase of the expression of c-Myc and Cyclin D1 was observed in MCF-7-TamR (Fig. 3c) and T47D-TamR cells (Fig. S3c). Meanwhile, ARPP-19 and ERRγ were increased greatly at both protein (Fig. 3d/S3d) and mRNA (Fig. 3e/S3e) levels in MCF-7-TamR and T47D-TamR cells compared with their parental cells respectively. These results indicated that miR-320a was negatively correlated with the expression of ARPP-19 and ERRγ, and their downstreams c-Myc and Cyclin D1 in tamoxifen-resistant ER+ breast cancer cells, which may relate to tamoxifen resistance.

### Restored miR-320a expression or knockdown of its targets re-sensitizes tamoxifen resistant breast cancer cells

Since miR-320a expression was low in TamR cell lines, we next asked whether the restoration of miR-320a expression or silencing of its targets could re-sensitize tamoxifen resistant breast cancer cells. miR-320a inhibited the cell viability (Fig. 4a and Fig. S5a) and soft agar colony formation (Fig. 4b and Fig. S5b) of MCF-7/T47D-TamR cells without tamoxifen treatment compared with negative control (NC), and on exposure to tamoxifen, overexpression of miR-320a re-sensitized TamR cells to tamoxifen. We next examined whether the knockdown of ARPP-19, ERRγ, c-Myc, and Cyclin D1 expression could restore tamoxifen sensitivity. Cell viability analysis and soft agar assay showed that si-ARPP-19, si-ERRγ, si-Myc, and si-Cyclin D1 (Fig. 4c) partially restored the sensitivity of the TamR cells to tamoxifen (Fig. 4d/S5c and 4e/S5d). To exclude that these effects are potentially due to off-target effects of the small interfering RNA (siRNA) pools used for gene silencing, we over expressed the ARPP-19, ERRγ, c-Myc, and Cyclin D1 plasmids to rescue the indicated siRNAs silencing efficacy(Fig. 4c). Hence, restoration of miR-320a expression or knockdown of its targets did indeed re-sensitize tamoxifen resistance breast cancer cells to tamoxifen *in vitro*.

### Forced expression of miR-320a re-sensitizes TamR breast cancer cells to tamoxifen in xenografts

**Figure 5:**
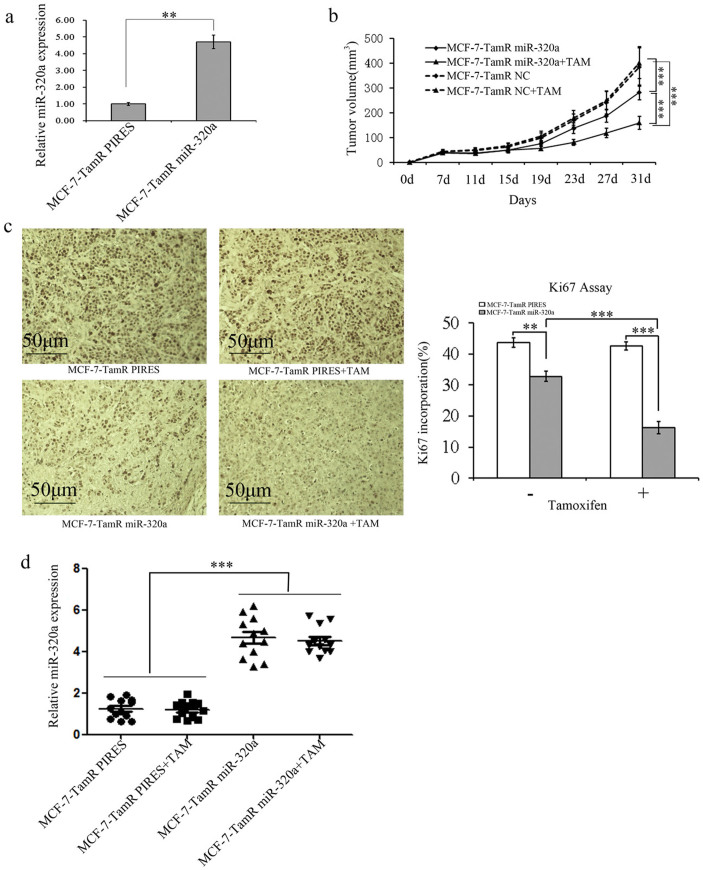
Forced expression of miR-320a re-sensitizes TamR breast cancer cells to tamoxifen in xenografts. (a). Forced expression of miR-320a in MCF-7-TamR cells was detected by real-time PCR. (b). Tumor growth curves derived from MCF-7-TamR and parental cells transplanted in nude mice in the presence or absence of tamoxifen supplement. Numerical data was expressed as mean ± SD of 6 mice. (c), Ki-67 staining of sections was used to examine the proliferation of the tumors formed. The quantified result was shown on the right. (d). The expression levels of miR-320a in the xenograft tumors were examined by real-time PCR. *P < 0.05, **P < 0.01, ***P < 0.001.

The roles of miR-320a in affecting the tamoxifen resistance of ER+ breast cancer cells were further investigated *in vivo*. MCF-7-TamR cells were stably transfected with miR-320a-expressing pIRESneo3- miR-320a plasmid (designated as MCF-7-TamR miR-320a) (Fig. 5a) or with the empty vector pIRESneo3 (designated as MCF-7- TamR PIRES). MCF-7-TamR miR-320a or MCF-7-TamR PIRES cells were implanted subcutaneously into the dorsal flank of 4-week old female nude mice. Both cell lines formed palpable tumors after less than 1 week and the volume of the tumors reached about 100 mm^3^ after 11 days, then the mice in both groups were randomized to be implanted subcutaneously with tamoxifen pellets or placebo pellets. Tumors derived from MCF-7-TamR miR-320a cells grew much slower than those from MCF-7-TamR PIRES cells. Moreover, in tamoxifen-treated groups, the tumors derived from MCF-7-TamR PIRES cells exhibited pronounced resistance to tamoxifen as expected, whereas the growth of MCF-7-TamR miR-320a derived tumors was significantly reduced by tamoxifen (Fig. 5b). Concordantly, the staining intensity and the number of hyperproliferative Ki-67 (Fig. 5c) were decreased in tumors that were derived from MCF-7-TamR miR-320a compared with those from MCF-7-TamR PIRES. MiR-320a levels in all groups of tumors that were examined after harvest (Fig. 5d). As a result, miR-320a reduced cell growth and enhanced the sensitivity to tamoxifen of ER+ breast cancer cells *in vivo*.

### c-Myc mediates progesterone(P_4_)and estrogen (E_2_)-regulated miR-320a expression

**Figure 6:**
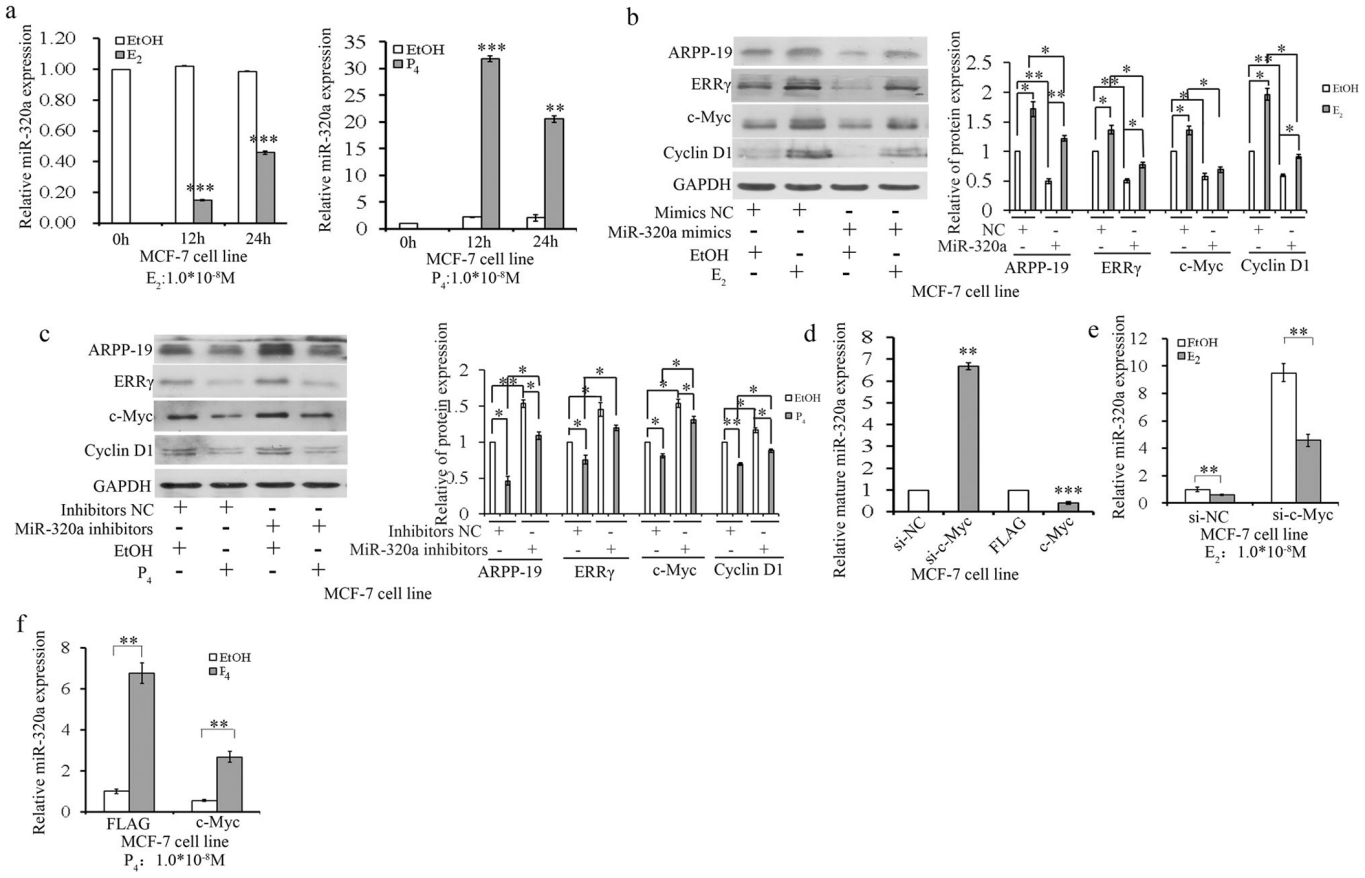
c-Myc mediates progesterone (P_4_) and estrogen (E_2_)-regulated miR-320a expression. (a). Real-time PCR analysis of miR-320a expression levels in MCF-7 cells treated with E_2_ or P_4_ for 0 h, 12 h, and 24 h. (b) and (c). Immunoblot analysis (the left panel) of ARPP-19, ERRγ, c-Myc, and Cyclin D1 levels in MCF-7 cells transfected with miR-320a mimics/inhibitors in the presence or absence of E_2_ (b) or P_4_ (c) treatment and the corresponding densitometric analysis (the right panel). (d). Real-time PCR analysis of miR-320a expression levels in MCF-7 cells transfected with either c-Myc specific siRNA or c-Myc-flag (c-Myc) overexpression plasmids. e and f. Real-time PCR analysis of miR-320a expression levels in MCF-7 cells transfected with si-c-Myc (e)/c-Myc overexpression plasmids (f) for 24 h and followed with stimulation with E_2_ (e)/P_4_ (f). Results are presented as an average of at least three replicates. *P < 0.05, **P < 0.01, ***P < 0.001.

*Atho*ugh the expression and function of miR-320a in TamR cells were first investigated in this study, further understanding of the regulation of miRNA expression would be helpful to gain insights into both normal and pathological conditions. There was a report to show that the expression of miR-320 was promoted by P_4_, but not visibly affected by E_2_ in the ovariectomized rat uterus^19^. Herein, mature miR-320a expression levels were significantly increased in MCF-7 or T47D cells treated with progesterone (P_4_), but were reduced by estrogen (E_2_) treatment (Fig. 6a and Fig. S6a). As previously reported, the expression of c-Myc mRNA decreased significantly in response to progesterone in chick oviduct^20^. To investigate whether P_4_ and E_2_ have an effect on the expression of ARPP-19, ERRγ, c-Myc, and Cyclin D1 through miR-320a, MCF-7 cells were transfected with miR-320a mimics or inhibitors and further treated with E_2_ or P_4_ for 12 h, respectively. Immunoblot data revealed that the expression of miR-320a target genes were downregulated by P_4_ treatment, an effect that could be rescued by miR-320a depletion (Fig. 6c). However, their expression increased in the E_2_-treated MCF-7 cells, but could be diminished by miR-320a mimics (Fig. 6b). In addition, the knockdown and overexpression of c-Myc increased and decreased mature miR-320a expression in MCF-7 and T47D cells, respectively (Fig. 6d and Fig. S6b). Furthermore, si-c-Myc rescued E_2_ repressed-miR-320a expression (Fig. 6e and Fig. S6c), while the overexpression of c-Myc decreased the expression of P_4_-promoted miR-320a in both MCF-7 and T47D cells (Fig. 6f and Fig. S6d). These results indicated that c-Myc mediated P_4 _and E_2_-regulated miR-320a expression in ER+ breast cancer cells and may relate with the acquisition of resistance to tamoxifen.

## Discussion

ERα positive breast cancer patients are often treated with selective estrogen-receptor modulators (SERMs) such as tamoxifen as the standard adjuvant therapeutic regimes. Many of the breast tumors that initially respond to tamoxifen therapy eventually develop resistance and recur^21^. The altered expression of specific microRNAs has been reported to contribute to tamoxifen resistance^22^, such as miR-221/222^11^, and miR-375^3^.

In this study, we developed and characterized an *in vitro* tamoxifen resistance model to investigate the potential roles of miRNAs in the acquisition of tamoxifen resistance in ER+ breast cancer. MiR-320a was remarkably reduced in TamR cells compared with their parental cells. Earlier work demonstrated that miR-320a was a critical target of PTEN in stromal fibroblasts that directly controlled ETS2 expression and instructed the tumor microenvironment to suppress the multiple aggressive phenotypes that are associated with advanced stages of breast cancer, including tumor-cell invasiveness and increased antigenic networks^23^. It was also observed that miR-320a suppressed human colon cancer cell proliferation by directly targeting β-Catenin and decreased significantly in the recurrence of stage II MSS cancer^24^. Meanwhile, we observed that forced expression of miR-320a in TamR cells inhibited cell growth, cell colony formation, tumor-initiating capacities in vivo, and restored sensitivity of the TamR cells to tamoxifen both *in vitro* and *in vivo*. In comparison, the depletion of miR-320a endowed the parental cells MCF-7 and T47D the resistance to tamoxifen. Moreover, we identified that miR-320a directly targeted ARPP-19 and ERRγ. In addition, miR-320a was regulated by c-Myc, suggesting a potential mechanism that was utilized by miR-320a to regulate tamoxifen resistance in breast cancer. Additionally, P_4_ might sensitize tamoxifen-resistant breast cancer cells to tamoxifen partially by boosting miR-320a expression. Thus, we have revealed a novel role of miR-320a in sensitizing tamoxifen-resistant breast cancer cells to tamoxifen, implying that the interference of miR-320a expression might be used as a potential approach to circumvent acquired tamoxifen resistance.

**Figure 7:**
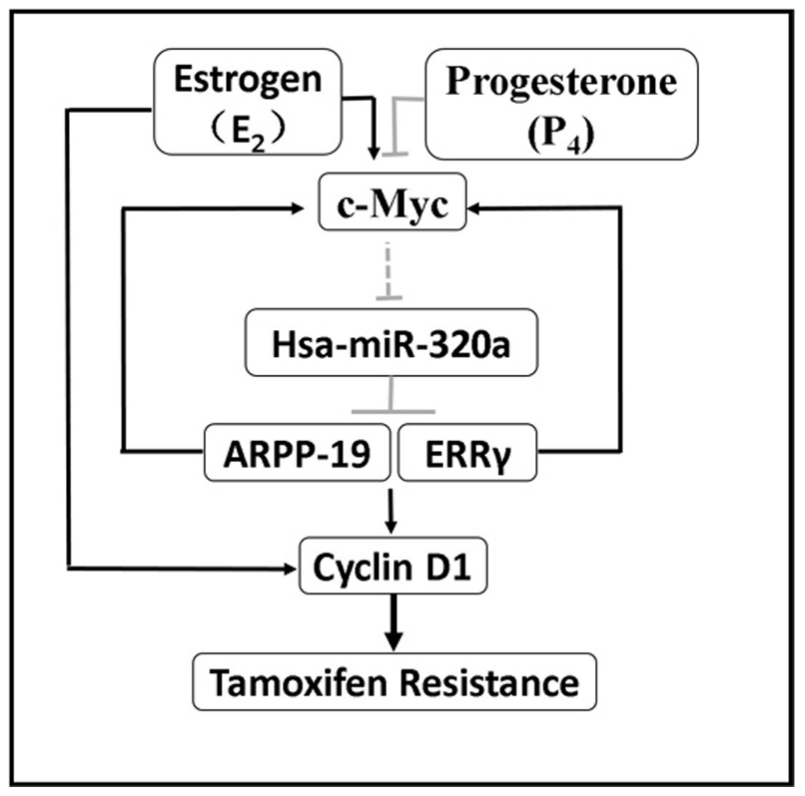
Proposed model for the critical roles of miR-320a in the mediation of tamoxifen sensitivity. P_4_ or E_2_ specifically promotes or represses miR-320a expression via c-Myc to modulate the expression of ARPP-19, ERRγ, and their downstreams c-Myc, Cyclin D1, which are also directly regulated by E_2_. MiR-320a further directly downregulates ARPP-19 and ERRγ, which in turn represses c-Myc expression, and subsequently re-sensitizes tamoxifen-resistant breast cancer cells to tamoxifen. A positive feedback loop is proposed as indicated between c-Myc and miR-320a to regulate tamoxifen resistance in breast cancer.

Elevated expression of c-Myc has been involved in disease progression and relapse of ER+ breast cancer patients that have been subjected to adjuvant hormonal therapy, presumably by promoting the proliferation and survival of ER+ breast cancer cells^25^. Further, c-Myc activation signature and high c-Myc expression levels were both predictive of poor outcomes following tamoxifen therapy^18^. The expression of c-Myc was precisely regulated by a complex signaling network at both transcriptional and post-transcriptional levels. Herein, our findings supplemented this network by presenting a novel positive feedback loop (Fig. 7). The presence of c-Myc repressed miR-320a expression and thereby indirectly enhanced the expression of ARPP-19 and ERRγ, which cooperatively increased Cyclin D1 expression. Depletion of ARPP-19 and ERRγ further reduced the expression of c-Myc. Our current findings are consistent with the report that Cyclin D1 is involved in the tamoxifen-resistant phenotype of ER+ breast cancer cells^26^,^27^. Thus, we have identified a positive feedback loop between c-Myc and the Cyclin D1 cascade.

Furthermore, estrogen elicited signaling has been reported to endow mammary epithelial cells with tamoxifen resistance, mainly by transcriptionally inducing c-Myc expression^22^. Progesterone was also reported to inhibit breast cancer cell growth and induced apoptosis^28^. Our results showed that miR-320a, upregulated by P_4_ through c-Myc, sensitized tamoxifen-resistant breast cancer cells to tamoxifen by targeting ARPP-19 and ERRγ. Thus we revealed a novel P_4_ elicited signaling cascade herein (P_4_-c-Myc-miR-320a- ARPP-19/ERRγ/c-Myc/Cyclin D1) to modulate tamoxifen-resistant breast cancer cells.

Collectively, we reported in this study that decreased expression of miR-320a could endow tumorigenic mammary epithelial cells with tamoxifen resistance *in vitro* and *in vivo* via the de-repression of ARPP-19 and ERRγ and the activation of the c-Myc and Cyclin D1 pathways. The c-Myc/miR-320a axis added a novel understanding of the mechanism of acquired tamoxifen resistance in breast cancer. The manipulation of miR-320a expression might represent an attractive approach to circumvent anti-estrogen resistance in ER+ breast cancer.

## Methods

### Tissue samples

58 fresh tissues including 31 breast cancer and 27 normal breast samples were obtained from patients that underwent surgery at the First Affiliated Hospital of Anhui Medical University between 2009 and 2010. All patients signed informed consent documents approving the use of their tissues for research purposes. All the experiments on live vertebrates were performed in accordance with the relevant guidelines and regulations. This study received ethical approval from the institutional review boards of the USTC and the Anhui Medical University.

### Vectors and oligonucleotides

The psiCHECK^TM^-2 dual luciferase reporter vectors which were constructed based on psiCHECK^TM^2 vector (Promega, Madison, WI) were kindly provided by Biliang Zhang (Guangzhou Institute of Biomedicine and Health, Chinese Academy of Sciences, China), as described by Yao *et al*.^13^. The c-Myc overexpression vector was kindly provided by Dr. Mian Wu (USTC, China) at the BamHI and XhoI sites. For construction of the luciferase reporter plasmids, wild type (WT) ARPP-19 3′UTR was obtained by amplifying a 930-bp 3′UTR fragment of ARPP-19 harboring the miR-320a-binding site predicted by TargetScan, whereas mutated (MT) ARPP-19 3′UTR was generated through PCR-based site-directed mutagenesis. WT and MT ARPP-19 3′ UTR, as well as full-length ERRγ 3′UTR sequences, were inserted into the XhoI and NotI sites of the psiCHECK-2 reporter vector, immediately downstream of the stop codon of an SV40 promoter-driven Renilla luciferase gene. The ARPP-19,Cyclin D1 and ERRγ expression constructs were generated by cloning the human cDNA into the p3XFLAG-myc-CMV™-24(FLAG) and pEGFP-C1(GFP) vector at the EcoRI and SalI sites, respectively. The miR-320a mimics are chemically synthesized, double-stranded RNAs that mimic mature endogenous miR-320a after transfection into cells and miR-320a mimics controls are non-human miRNAs, predicted to not target the human genome/transcriptome, whereas the miR-320a inhibitors are single stranded 2-O-methyl-modified oligoribonucleotide fragments exactly antisense to miR-320a and miR-320a inhibitors controls are random sequences anti-miR-320a molecule that produce no identifiable effects on miR-320a function. The sequence of miR-320a mimics and miR-320a inhibitors, as well as their negative controls (NCs), are as follows:

miR-320a mimics:5′AAAAGCUGGGUUGAGAGGGCGA3′;

miR-320a inhibitors:5′UCGCCCUCUCAACCCAGCUUUU3′;

miR-320a mimics negative control:5′UUCUCCGAACGUGUCACGUTT3′,

miR-320a inhibitors negative control:5′CAGUACUUUUGUGUAGUACAA3′;

which were synthesized and purified by Shanghai Gene-Pharma Co. (Shanghai, China). The siRNAs (si-ARPP-19, si-ERRγ, si-Cyclin D1, si-Myc) were obtained from Santa Cruz Biotechnology Inc. (Santa Cruz, CA, USA).

### Cell culture and transfection

The human mammary carcinoma cell lines MCF-7 and T47D were obtained from the American Type Culture Collection (Rockville, MD, USA) and cultured as recommended. We derived MCF-7 and T47D cell lines and exposed them to tamoxifen (1 μM) for >12 months (designated MCF-7-TamR and T47D-TamR, respectively) as described previously. ERα expression was significantly decreased in both MCF-7-TamR and T47D-TamR cell lines compared with their parental cells^21^, indicating that ERα may not be functional in TamR cells. All cells were maintained in a humidified incubator at 37°C and 5% CO_2_. For induction experiments using estrogen (E_2_) or progesterone (P_4_), cells were cultured for at least 3 days in phenol red-free RPMI-1640 with 5% dextran-coated charcoal-treated serum before treatment. MCF-7/MCF-7-TamR and T47D/T47D-TamR cell lines were maintained in RPMI 1640 with 25 mmol/L HEPES (Invitrogen/Life Technologies Inc., Grand Island, NY). The medium was supplemented with 10% fetal bovine serum (FBS; Invitrogen/Life Technologies) and 1% penicillin-streptomycin (Invitrogen/Life Technologies) for all cell lines. The medium for MCF-7-TamR and T47D-TamR cell lines was supplemented with 1 μM tamoxifen.

### Luciferase reporter assay

Cells were co-transfected with either 60 pmol miR-320a mimics or 80 pmol miR-320a inhibitors and 200 ng psiCHECK-2 vectors in 24-well plates with three replicate wells using Lipofectamine 2000 (Invitrogen/Life Technologies) for each condition. Luciferase activity was measured 30 h after transfection using the Dual Luciferase Reporter Assay System (Promega Corp.) with a luminometer, according to the manufacturer's instructions. Renilla luciferase activity was normalized to Firefly luciferase activity for each transfected well.

### Western blotting

Cells were lysed in RIPA buffer (50 mM Tris-HCl, pH 7.4, 150 mM NaCl, 1% Triton X-100, 1% sodium dodecyl sulfate, 1% sodium deoxycholate, 1 mM EDTA) together with a complete (EDTA-free) protease inhibitor cocktail (Kodak, Rochester, NY, USA), 1 mM phenylmethylsulfonyl fluoride, and phosphatase inhibitors (5 mM sodium orthovanadate). Protein lysates were resolved by SDS-PAGE, transferred to Hybond enhanced chemiluminescence (ECL) Nitrocellulose membranes (Amersham Biosciences, Freiburg, Germany), immunoprobed with antibodies, and visualized by ECL detection reagents (Kodak, Rochester, NY, USA). The primary antibodies of ARPP-19 (sc-135145), ERRγ (sc-133561), Cyclin D1 (sc-753) and c-Myc (#9402) were purchased from Santa Cruz Biotechnology or Cell Signaling. As described in Liu *et al.*^29^ *and* Yin M *et al.*^14^, protein levels were normalized to GAPDH and quantified using Tanon Gel image system (Tanon, Shanghai, China).

### Cell proliferation assays

Cell proliferation was measured using the cell counting kit-8 (CCK-8) (DojindoLaboratories, Kumamoto, Japan). MCF-7/T47D-TamR cells were seeded in full growth medium. After 24 h, the medium was replaced with RPMI-1640 only, then 150 nM miR-320a mimics (and a negative control) were transfected using Lipofectamine 2000. After 48 h, 3000 cells in a volume of 100 μL were seeded in a 96-well plate for 24 h and then replaced by 200 μl full growth medium with 1 μmol/L tamoxifen for 5 days. Each treatment had five replicates on the plate. After the assay began, 10 μl of CCK-8 solution was added to the medium and then incubated at 37°C for 2 h. Cell numbers were estimated by measuring the absorbance at 450 nm using a 96-well format plate reader (ELx800 Universal Microplate Reader; Biotek Instrument Inc., Highland Park, VT, USA).

### Soft agar colony formation assays

Five thousand cells were seeded in 1.5 mL medium supplemented with 0.35% agarose, on a 1.5 mL base of medium with 0.5% agarose. Then 2 mL of normal liquid medium was added on top of the 1.5 mL medium containing 0.35% agarose and cells. For tamoxifen-treatment, tamoxifen was added in the upper 2 mL of liquid medium. Soft agar assays were performed in six-well plates and in triplicate. The number of colonies was counted (and images taken) after 10 days.

### Real-time PCR assay

For real-time PCR assays, total RNA was extracted from cultured cells using Trizol (Invitrogen). The cDNA was synthesized from 500 ng of purified RNA using a PrimeScript RT reagent kit (TaKaRa Bio, Inc., Otsu, Japan) according to the manufacturer's instructions. Real-time PCR was performed in an Applied Biosystems Step One real time PCR system using a SYBR Premix Ex Taq II Kit (Takara Bio, Inc., Shiga, Japan). Each sample was analyzed in triplicate and the experiment was repeated three times. The primers for ARPP-19, ERRγ and β-actin are as follows:

ARPP-19 Forward: 5′GCCTGGAGGTTCAGATTT 3′,

Reverse: 5′CAGTAGGAAGTTGCTTGTTC3′;

ERRγ Forward: 5′TGACACTGGCAAAACAATGCA3′,

Reverse: 5′GGTCCTTTTCACCAGCAAGCT3′;

β-actin Forward: 5′TGGCACCCAGCACAATGAA3′,

Reverse: 5′CTAAGTCATAGTCCGCCTAGAAGC3′.

PCR conditions were as follows: 95°C for 30 sec, followed by 40 cycles at 95°C for 5 sec, 60°C for 34 sec, and 95°C for 15 sec, 60°C for 1 min, and 95°C for 15 sec, as described previously^13^,^30^. Expression levels were normalized to β-actin expression. The mature miR-320a levels were measured by TaqMan® MicroRNA Assays (Applied Bio systems, Foster City, CA) after transfection with miR-320a mimics/inhibitors into the cells. PCR conditions were as follows: 95°C for 10 min, followed by 40 cycles at 95°C for 15 sec and 60°C for 1 min. Data was analyzed by using the comparative C_T_ method^31^, and the expression levels of miR-320a were normalized to the endogenous control U6 small nuclear RNA (snRNA).

### Xenograft analysis

All animal work was done in accordance with the protocol approved by the Institutional Animal Care and Ethics Committee of The University of Science and Technology of China. 82 bp DNA fragments corresponding to pre-miR-320a were cloned into the mammalian expression vector pIRESneo3 (Invitrogen). This was designated pIRESneo3-miR-320a. PIRESneo3-miR-320a (designated as MCF-7-TamR miR-320a) cells and the empty vector pIRESneo3 (designated as MCF-7-TamR PIRES) cells were suspended in PBS (500 × 104 per 125 μl per site), and injected subcutaneously into 4-week-old BALB/c nu/nu mice (Shanghai Slaccas Co, Shanghai, China). E_2_ pellets (Innovative Research of America, Sarasota, 0.18 mg; 60-day release) were subcutaneously implanted into the back of mice 1 day before cell inoculation. Tumors formed obviously about a week later. 11 days after cell-injection, tumors had reached about 100 mm^3^, and the mice in both groups were randomized to implant either Tamoxifen pellets (Innovative Research of America, Sarasota, 5 mg; 60-day release) or placebo pellets (as a control). The sizes of the tumors were measured every 4 days. The tumor growth curves were analyzed by measuring tumor length (L) and width (W), and the tumor volume was calculated based on the formula: Volume (mm^3^) = L × W^2^ × Π/6, as described previously^32^. Tumors were harvested 31 days after cell-injection and histological sections were taken. Immunohistochemical analysis was performed by using an UltraSensitive-SP kit (Maixin-Bio, Fuzhou, China) with a mouse polyclonal antibody against Ki-67 (Zhongshan Goldenbridge Biotechnology Co, Beijing, China), which is essentially a marker for cell proliferation. In order to quantitate the proportion of Ki-67 positive cells in each of the xenografting group, first, 6 fields of vision were randomly selected from every xenografting tumor sample; the second is to count the total cell and the Ki-67 positive cell to analyse the positive cell ratio; and then calculate the average value of every group as a measure of relative cell proliferation.

### Statistical analysis

Results are shown as the average and standard deviation (SD) of at least three biological and two technical replicates. *P* values, *P < 0.05, **P < 0.01 and ***P < 0.001 were determined by a 2-sided *t*-test, one-way ANOVA and two-way ANOVA. The expression of miR-320a in normal breast tissue, breast cancer, and in breast tumors stably expressing miR-320a was analyzed by PrismDemo.exe.

## Author Contributions

M.L. maintained all of the cell cultures, designed the siRNA experiment, ran qRT-PCR and western blots, performed the luciferase assays, was involved in the design of the study, and wrote the manuscript. K.D. provided all of the plasmids used in the study, performed the MTT and Soft Agar assays, performed the *in vivo* study, participated in the design of the study, and wrote the manuscript. G.Z., M.Y. and G.Y. were involved in the RNA and protein study, and participated in the writing of the manuscript. H.T. and J.L. participated in the *in vivo* study and critically revised the manuscript. L.L. and M.L. participated in data collection and were involved in the manuscript writing. T.Z. participated in data analysis and provided a critical review of the manuscript. F.S. conceived of the ideas contained in the manuscript, and critically revised the manuscript. All authors approved the final manuscript for publication.

## Supplementary Material

Supplementary Information Supplementary Information
