# Supplementary material for: MicroRNA-320a sensitizes tamoxifen-resistant breast cancer cells to tamoxifen by targeting ARPP-19 and ERRγ*
Source: Sci Rep. 2015 Mar 4;5:8735. doi: 10.1038/srep08735 (PMC4348640; doi:10.1038/srep08735)
Supplement: Supplementary Information [file srep08735-s1.pdf]

Supplementary information

MicroRNA-320a sensitizes tamoxifen-resistant breast cancer cells to tamoxifen by targeting ARPP-19 and ERRγ*

Mingrong Lü [a,b,#], Keshuo Ding [a,b,c,#], Guofeng Zhang[d], Mianmian Yin [a,b], Guidong Yao[e], Hui Tian [a,b], Jie Lian [a,b], Lin Liu [a,b], Meng Liang [a,b], Tao Zhu [a,b], Fei Sun [a,b]

Supplementary Figure S1

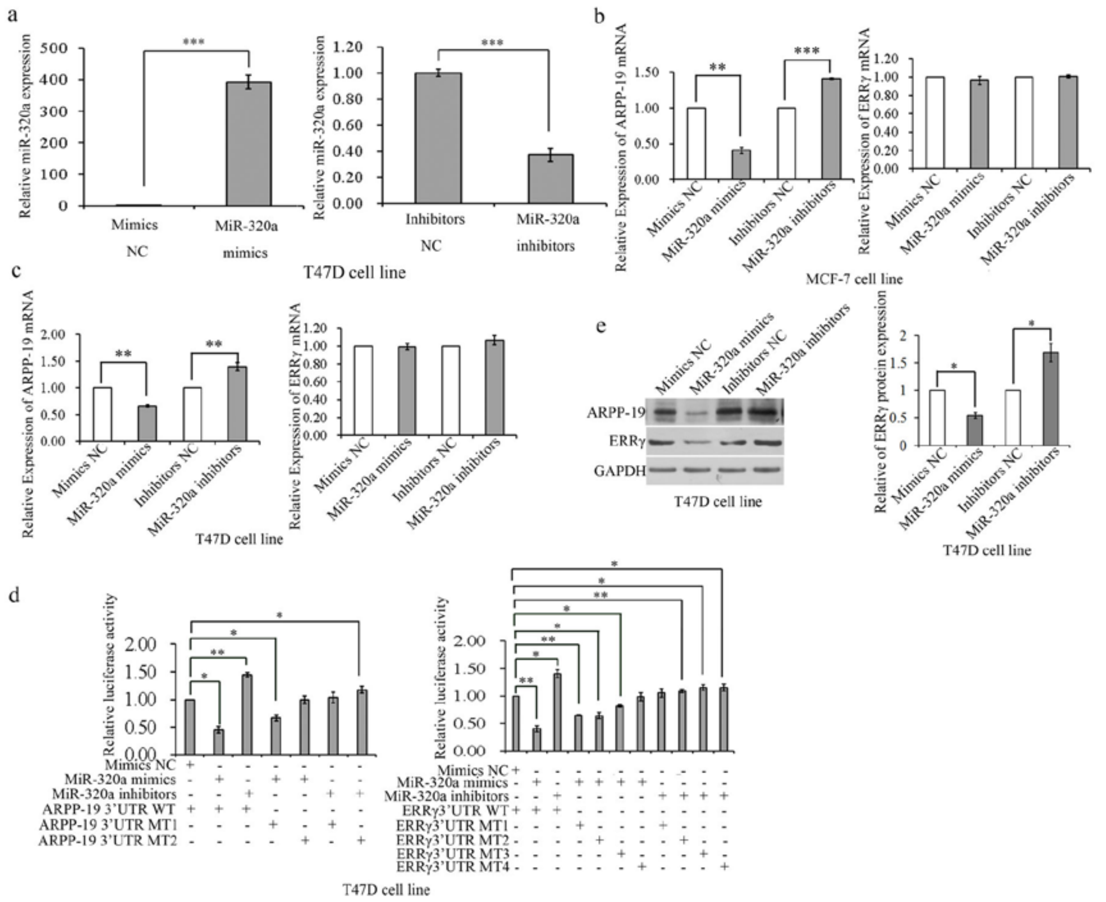

Figure S1. miR-320a directly targets ARPP-19 and ERRγ in T47D cell line. a. Real-time PCR analysis of miR-320a expression in T47D cells when transfected with miR-320a mimics or inhibitors. b and c. The expression levels of ARPP-19 and ERRγ mRNA were examined by real time PCR after transfected with miR-320a mimics or inhibitors for 48h in MCF-7 and T47D cells. d. Luciferase activity of luciferase gene fusing with wild-type or mutant 3'UTRs of ARPP-19 or ERRγ were measured after co-transfection with miR-320a mimics or inhibitors in T47D cell line. The luciferase activity was normalized with firefly luciferase activity. e. Western blot analysis of the expression levels of ARPP-19 and ERRγ in T47D cells transfected with miR-320a mimics or inhibitors and the densitometric analysis of ERRγ protein levels. Results are presented as an average of at least three replicates. *P < 0.05, **P < 0.01, ***P < 0.001.

Supplementary Figure S2

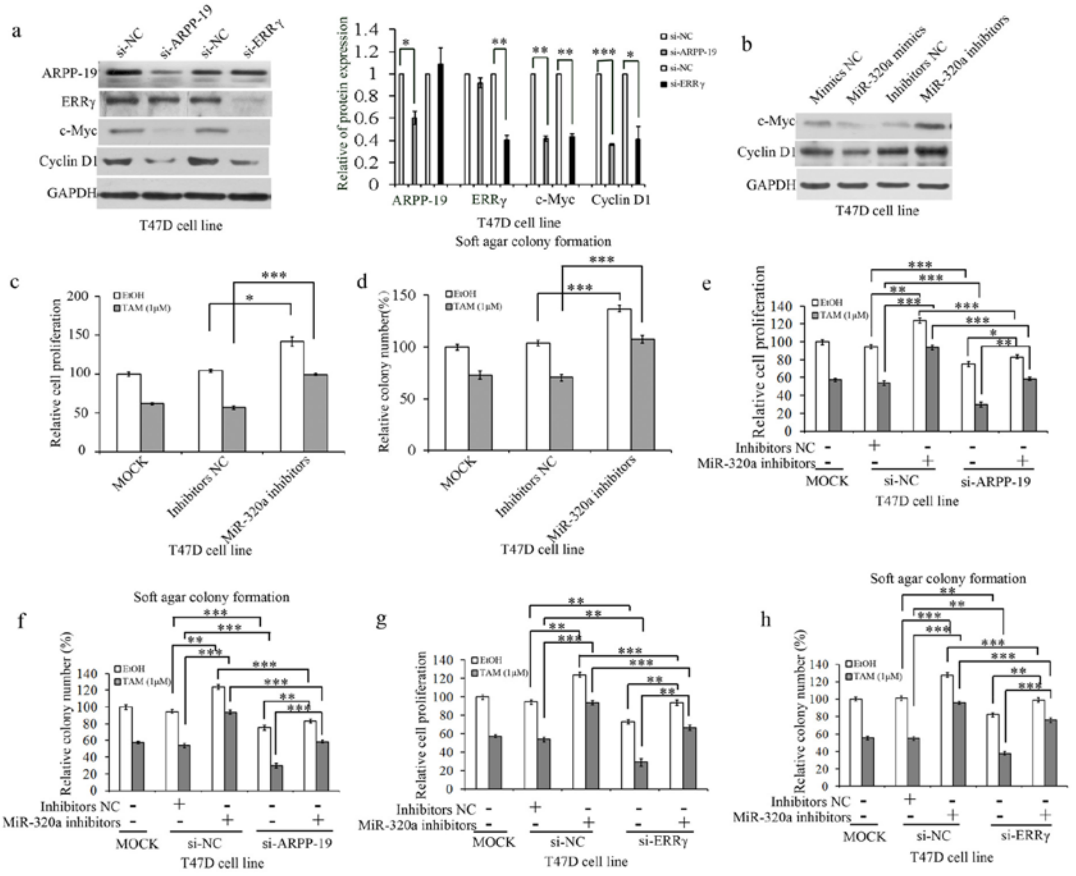

Figure S2. Knockdown of miR-320a reduced sensitivity of tamoxifen in T47D cells. a. Western blot analysis of ARPP-19, ERRγ, c-Myc, and Cyclin D1 expression levels in T47D cells transfected with si-ARPP-19 and si-ERRγ and the corresponding densitometric analysis . b. Western blot analysis of c-Myc and Cyclin D1 expression levels in T47D cells transfected with miR-320a mimics / inhibitors. c and d. Cell viability (c) and soft agar colony formation (d) analysis of T47D cells transfected with miR-320a inhibitors. e and f. Cell viability (e) and soft agar colony formation (f) analysis of T47D cells transfected with si-ARPP-19 and miR-320a inhibitors and co-transfected with si-ARPP-19 and miR-320a inhibitors on exposure to tamoxifen or not. f and g. Cell viability (f) and soft agar colony formation (g) analysis of T47D cells transfected with si-ERRγ and miR-320a inhibitors and co-transfected

with si-ERRγ and miR-320a inhibitors on exposure to tamoxifen or not. Results are presented as an average of at least three replicates. *P < 0.05, **P < 0.01, ***P < 0.001.

Supplementary Figure S3

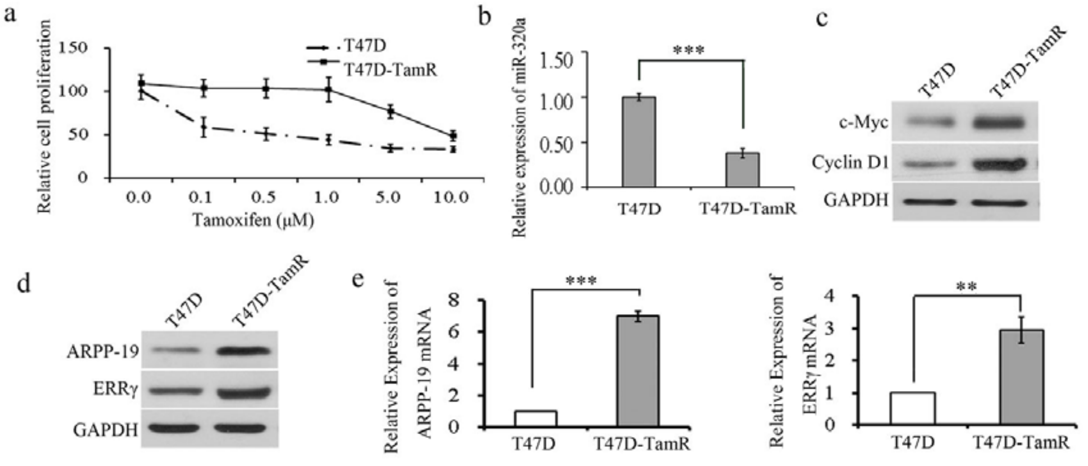

Figure S3. Tamoxifen resistant cell lines had a low level of miR-320a and a high level of c-Myc, Cyclin D1, ARPP-19, and ERRγ. a. Cell viability analysis of T47D-TamR cells as well as its parental cells treated with different doses of tamoxifen. b. Real-time PCR analysis of miR-320a expression in TamR cells compared with parental cells. c and d. Western blot analysis of the expression of c-Myc, Cyclin D1, ARPP-19 and ERRγ in T47D-TamR cells as well as parental cells. e. Real-time PCR analysis of ARPP-19 and ERRγ mRNA expression in TamR cells compared with parental cells. Results are presented as an average of at least three replicates. **P < 0.01, ***P < 0.001.

Supplementary Figure S4

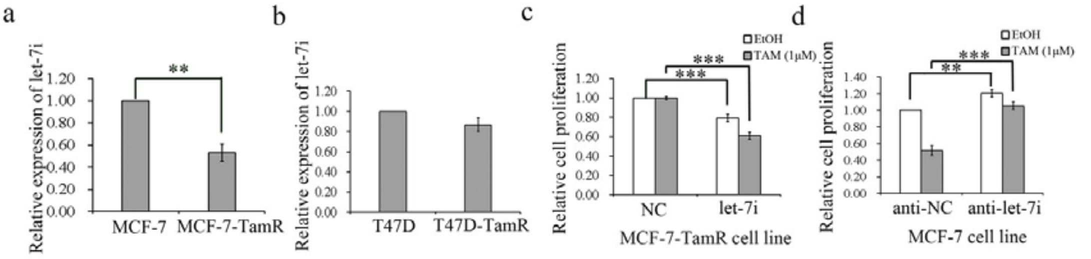

Figure S4.The expression of let-7i is reduced in MCF-7-TamR cells compared with MCF-7cell but not changed in T47D cells compared with parental cells and re-expression of let-7i in MCF-7-TamR cells sensitizes cells to tamoxifen while knock-down of let-7i promotes resistance to tamoxifen in WT cells. a and b, Real-time PCR analysis of let-7i expression in TamR cells compared with their parental cells. c and d, Cell viability analysis of MCF-7-TamR cells transfected with let-7i mimics and transfected with let-7i inhibitors in MCF-7 cells. Results are presented as an average of three replicates. $^{**}P < 0.01$, $^{***}P < 0.001$, determined by one-way ANOVA.

Supplementary Figure S5

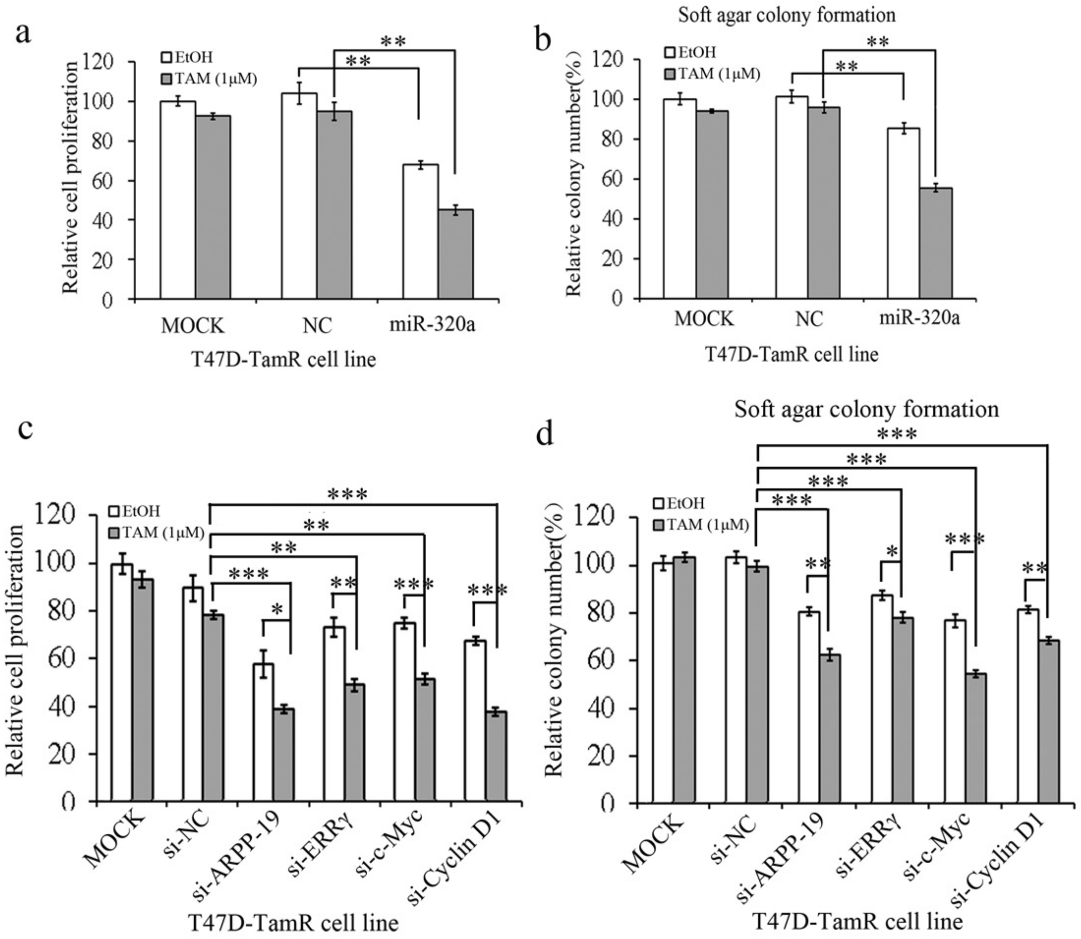

Figure S5. Re-expression of miR-320a or knockdown of its targets in TamR cells sensitizes these cells to tamoxifen. a and b. Cell viability (a) and soft agar colony formation (b) analysis of TamR cells transfected with miR-320a mimics. c and d. Cell viability (c) and soft agar colony formation (d) analysis of TamR cells transfected with si-ARPP-19, -ERRγ, -c-Myc, and -Cyclin D1. Results are shown as the average and s.d. of at least three biological and two technical replicates each,*P < 0.05, **P < 0.01, ***P < 0.001.

Supplementary Figure S6

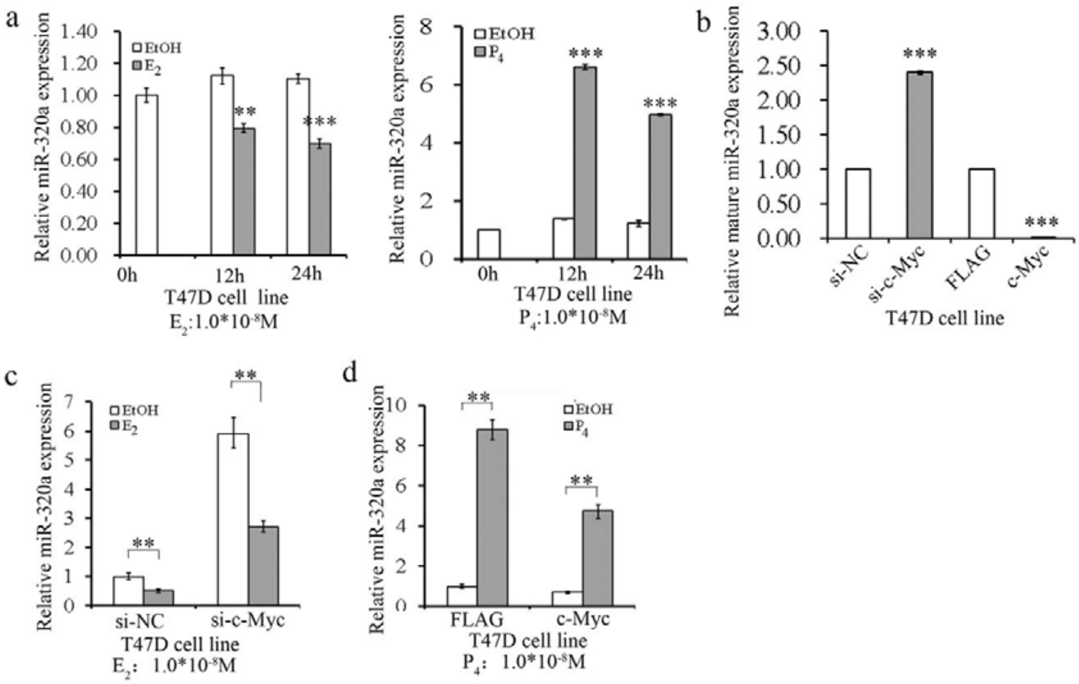

Figure S6. c-Myc mediates progesterone (P_4) and estrogen (E_2)-regulated miR-320a expression. a. Real-time PCR analysis of miR-320a expression levels in T47D cells treated with E_2 or P_4 for 0 h, 12 h, and 24 h. b. Real-time PCR analysis of miR-320a expression levels in T47D cells transfected with either c-Myc specific siRNA or c-Myc-flag (c-Myc) overexpression plasmids. c and d. Real-time PCR analysis of miR-320a expression levels in T47D cells transfected with si-c-Myc (c)/ c-Myc overexpression plasmids (d) for 24h and followed with stimulation with E_2 (c)/ P_4 (d). Results are presented as an average of at least three replicates. *P < 0.05, **P < 0.01, ***P < 0.001.

Supplementary Table 1 Expression of the indicated proteins or miR-320a in 31 breast cancer samples using immunohistochemistry (IHC) or real-time PCR.

Expression of the indicated proteins	ER+/PR+	ER+/PR-	ER-/PR+	ER-/PR-
HER2:-/+	13	0	1	2
	7↑ 6↓		1↓	1↑ 1↓
HER2:2+/3+	7	2	1	5
	5↑ 2↓	2↓	1↓	2↑ 3↓

↑ and ↓ mean that the miR-320a expression level in indicated samples was higher and lower than the average expression level of breast cancer samples. ER, estrogen receptor; PR, progesterone receptor; HER2, human epidermal growth factor receptor 2. The results of the IHC about HER2 test can be: 0 (negative), 1+ (also negative), 2+ (borderline), or 3+ (positive — HER2 protein overexpression).

Supplementary Table 2

(1) The proliferation of MCF-7/T47D cells transfected with indicated RNA on exposure to tamoxifen.

RNA / TAM / Cell line	INC si-NC	miR-320a I si-NC	INC si-ARPP19	miR-320a I si-ARPP19
MCF-7	0.56	0.89	0.28	0.53
P value		2.11965E-04	3.16781E-04	6.94745E-04<br>2.29533E-03
T47D	0.54	0.94	0.30	0.59
P value		7.10338E-04	2.6966E-04	5.2954E-04<br>2.07726E-03

(2) The colony formation of MCF-7/T47D cells transfected with indicated RNA on exposure to tamoxifen.

RNA / TAM / Cell line	INC si-NC	miR-320a I si-NC	INC si-ARPP19	miR-320a I si-ARPP19
MCF-7	62.3901801	96.8365894	30.12478176	69.77155352
P value		4.6699E-07	9.47434E-06	4.9387E-06<br>2.87916E-05
T47D	54.96031746	96.03174603	31.07936508	64.28571429
P value		8.21095E-09	3.99174E-7	8.45861E-09<br>3.07566E-05

(3) The proliferation of MCF-7/T47D cells transfected with indicated RNA on exposure to tamoxifen.

RNA / TAM / Cell line	INC si-NC	miR-320a I si-NC	INC si-ERRγ	miR-320a I si-ERRγ
MCF-7	0.56	0.89	0.37	0.69
P value		2.11965E-04	8.1651E-03	5.64645E-04<br>6.69833E-05
T47D	0.54	0.94	0.29	0.67
P value		7.10338E-04	8.4916E-03	7.1957E-04<br>3.87126E-03

(4) The colony formation of MCF-7/T47D cells transfected with indicated RNA on exposure to tamoxifen.

RNA / TAM / Cell line	INC si-NC	miR-320a I si-NC	INC si-ERRγ	miR-320a I si-ERRγ
MCF-7	62.3901801	96.8365894	33.71880632	76.89809557
P value		4.6699E-07	8.8611E-03	6.5874E-05<br>9.19273E-05
T47D	54.9603175	96.03174603	37.86507937	75.79365079
P value		8.21095E-09	8.4916E-03	2.11E-05<br>1.85256E-04

(1-4) The proliferation and the colony formation of MCF-7/T47D cells transfected with indicated RNA on exposure to tamoxifen. INC, inhibitor negative control; miR-320aI, microRNA-320a inhibitors. TAM, tamoxifen; The p value of each group: miR-320a I/si-NC p value, INC/si-NC vs miR-320a I/si-NC ; INC/si-RNA p value, INC/si-NC vs INC/si-RNA; miR-320a I/ si-RNA p value(up), miR-320a I/si-NC vs miR-320a I/ si-RNA; miR-320a I/ si-RNA p value(below), INC/si-RNA vs miR-320a I/ si-RNA.

Fig.1e

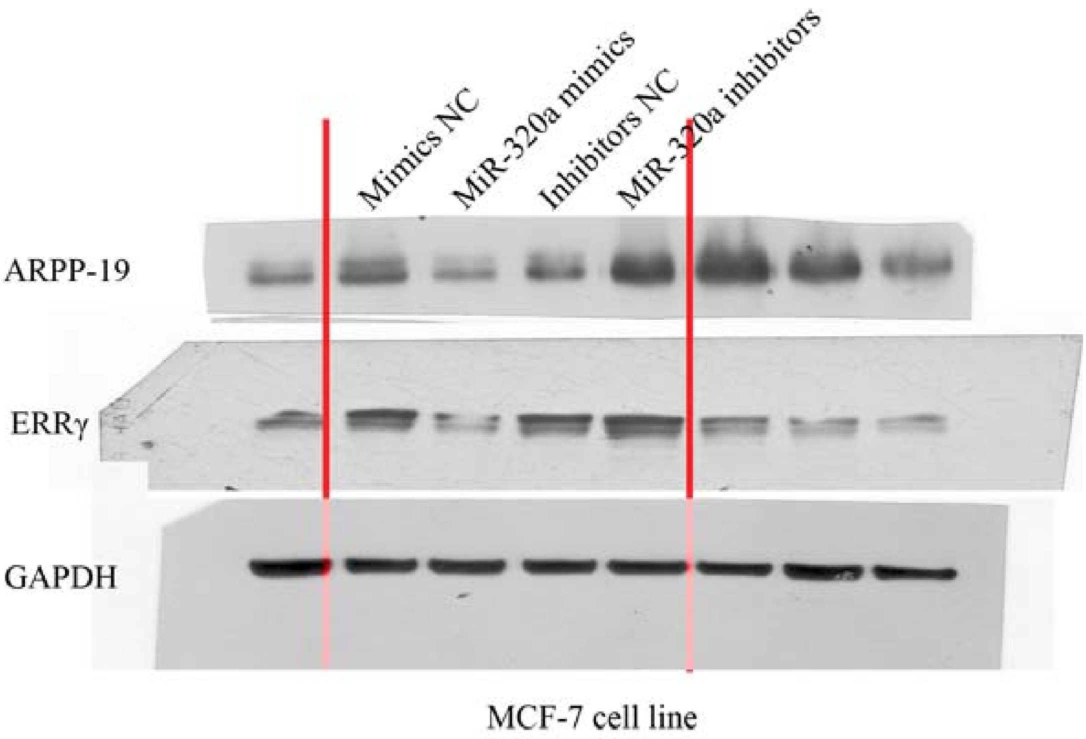

Western blot analysis of the expression levels of ARPP-19 and ERRγ in MCF-7 cells transfected with miR-320a mimics or inhibitors.

Fig.S1e

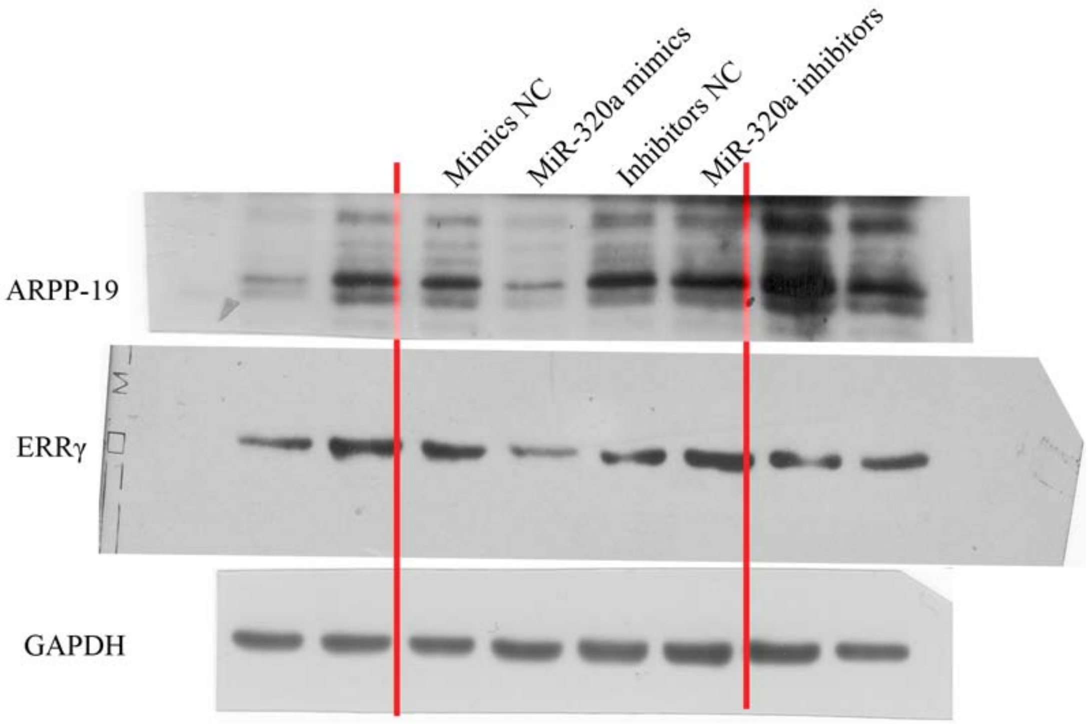

Western blot analysis of the expression levels of ARPP-19 and ERRγ in T47D cells transfected with miR-320a mimics or inhibitors.

Fig.2a

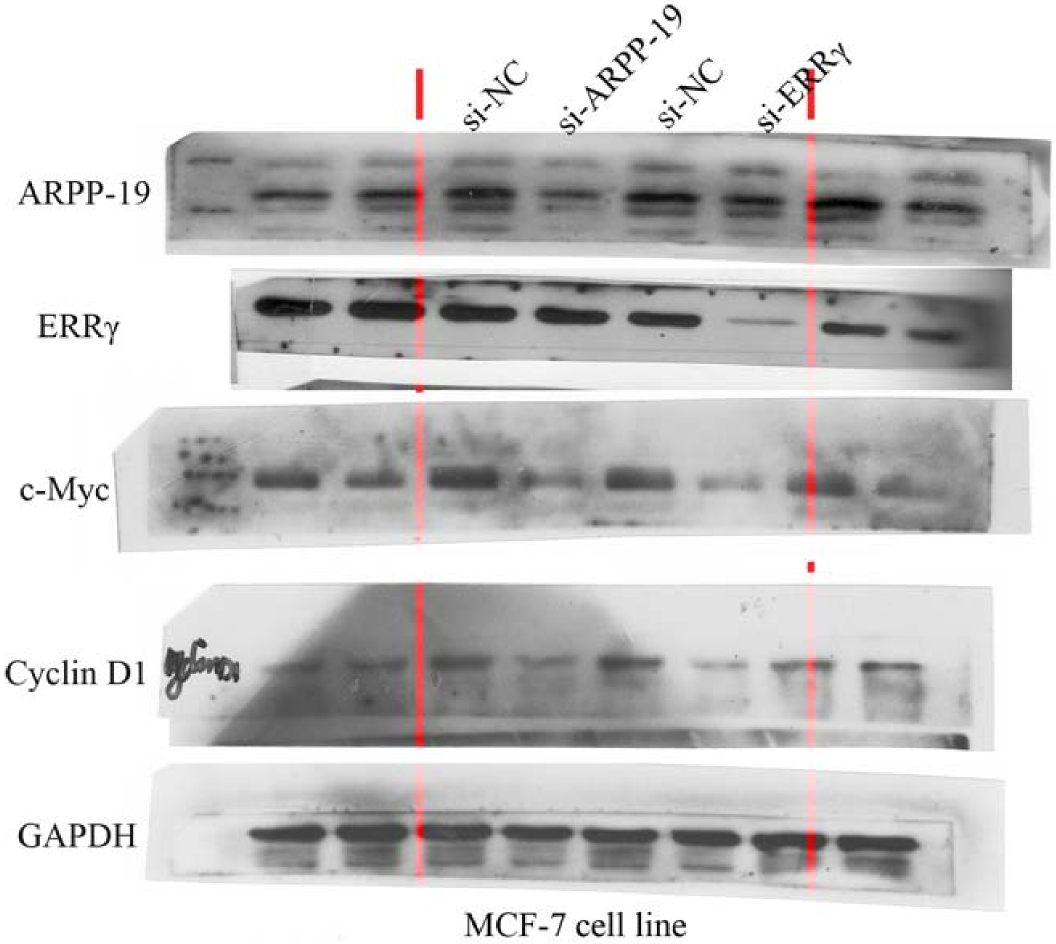

Western blot analysis of ARPP-19, ERRγ, c-Myc, and Cyclin D1 expression levels in MCF-7 cells transfected with si-ARPP-19 and si-ERRγ.

Fig.S2a

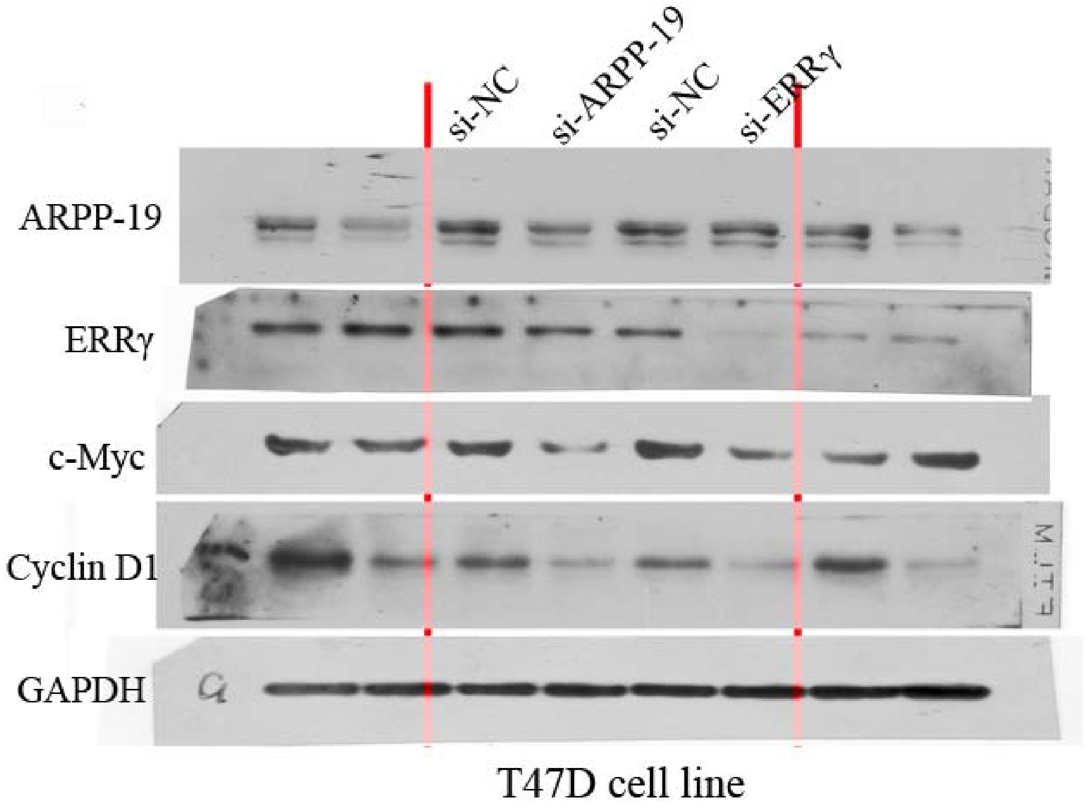

Western blot analysis of ARPP-19, ERRγ, c-Myc, and Cyclin D1 expression levels in T47D cells transfected with si-ARPP-19 and si-ERRγ.

Fig.2b

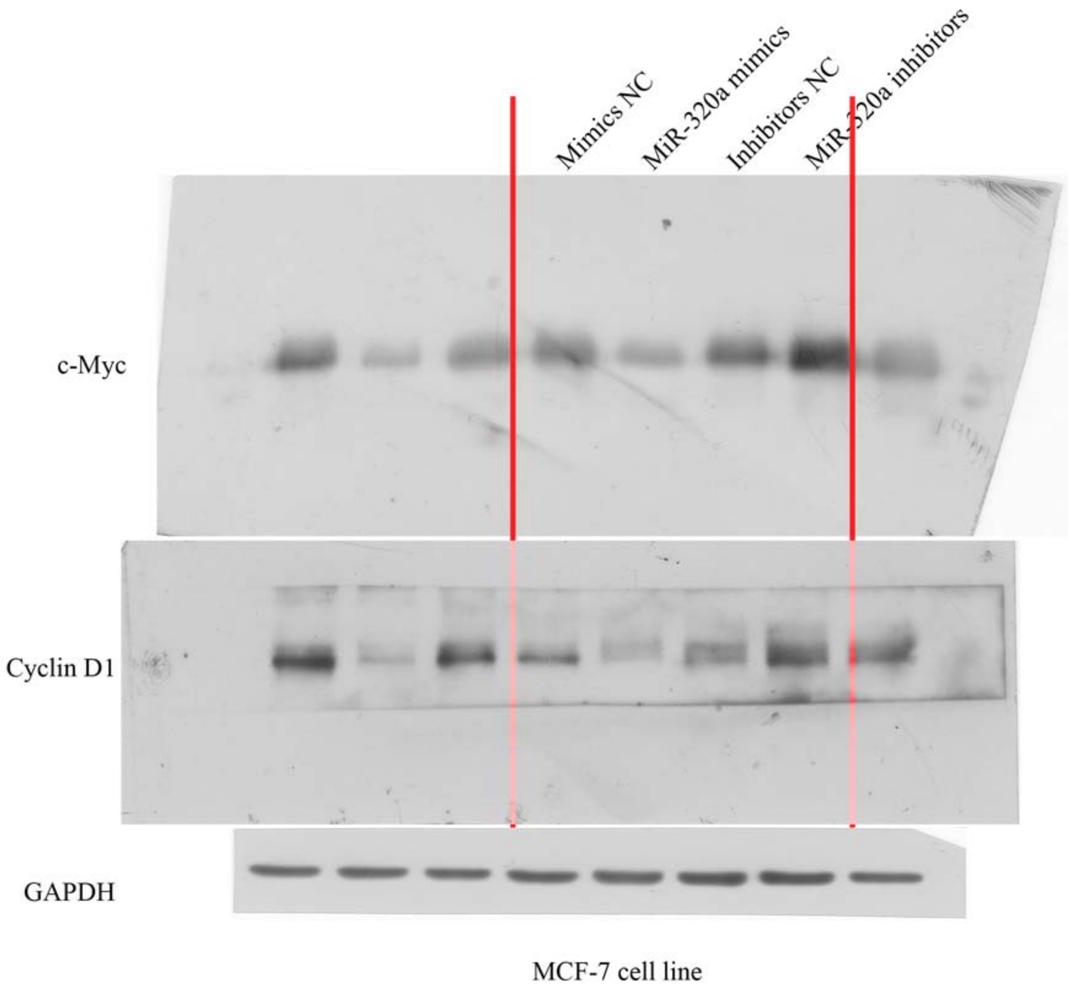

Western blot analysis of c-Myc and Cyclin D1 expression levels in MCF-7 cells transfected with miR-320a mimics / inhibitors.

Fig.S2b

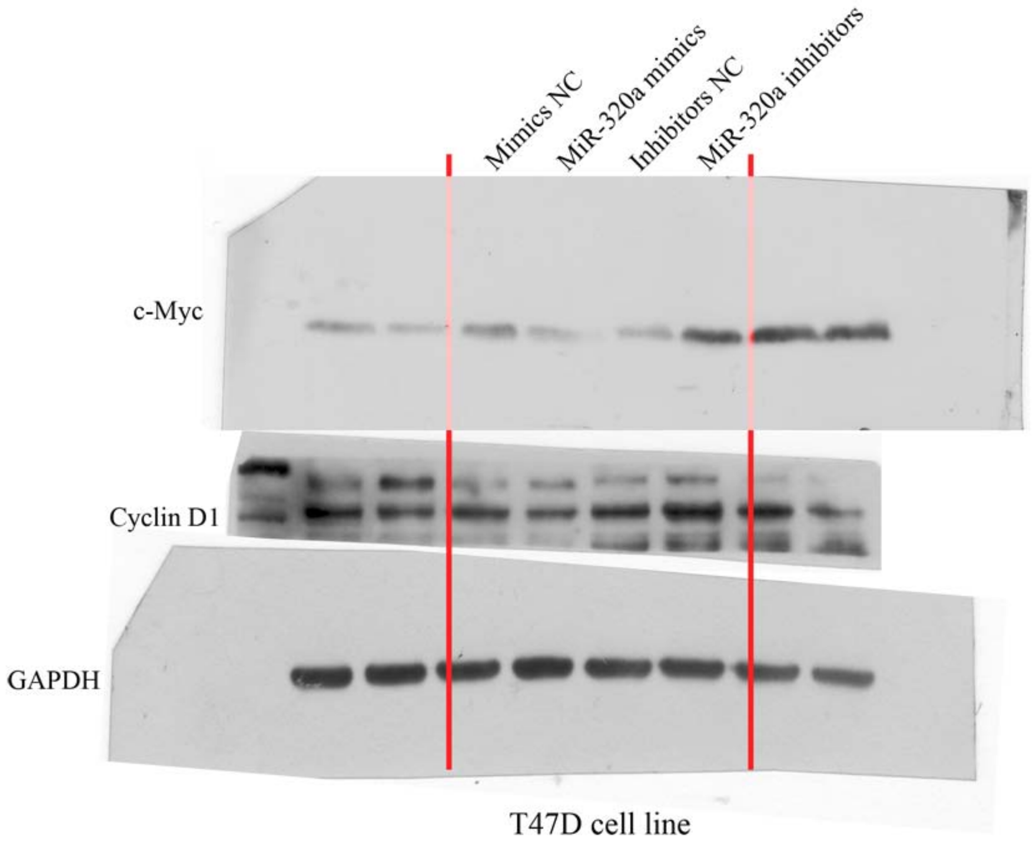

Western blot analysis of c-Myc and Cyclin D1 expression levels in T47D cells transfected with miR-320a mimics / inhibitors.

Fig.6b

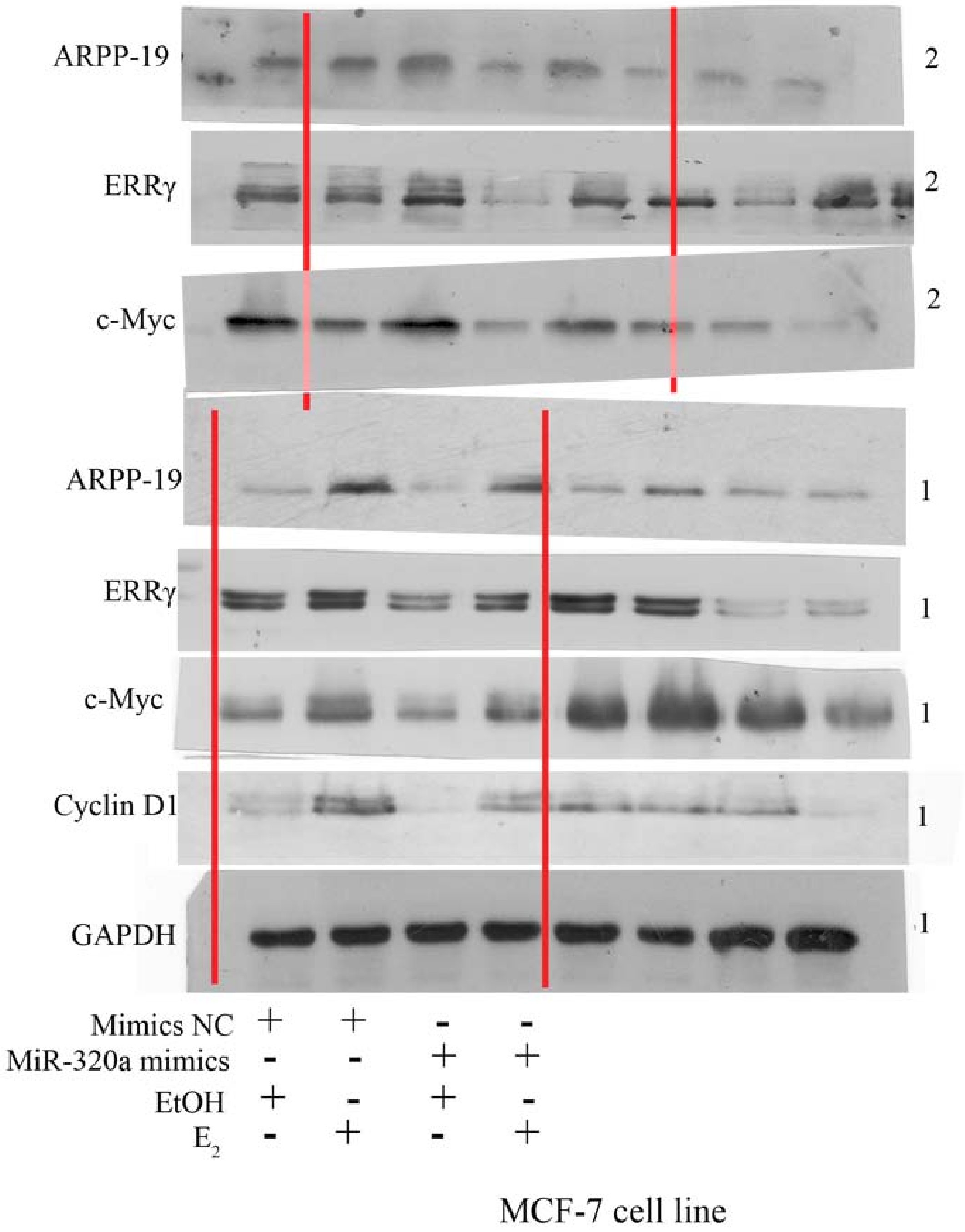

Immunoblot analysis of ARPP-19, ERRγ, c-Myc, and Cyclin D1 levels in MCF-7 cells transfected with miR-320a mimics in the presence or absence of E_2 treatment.

Fig.6c

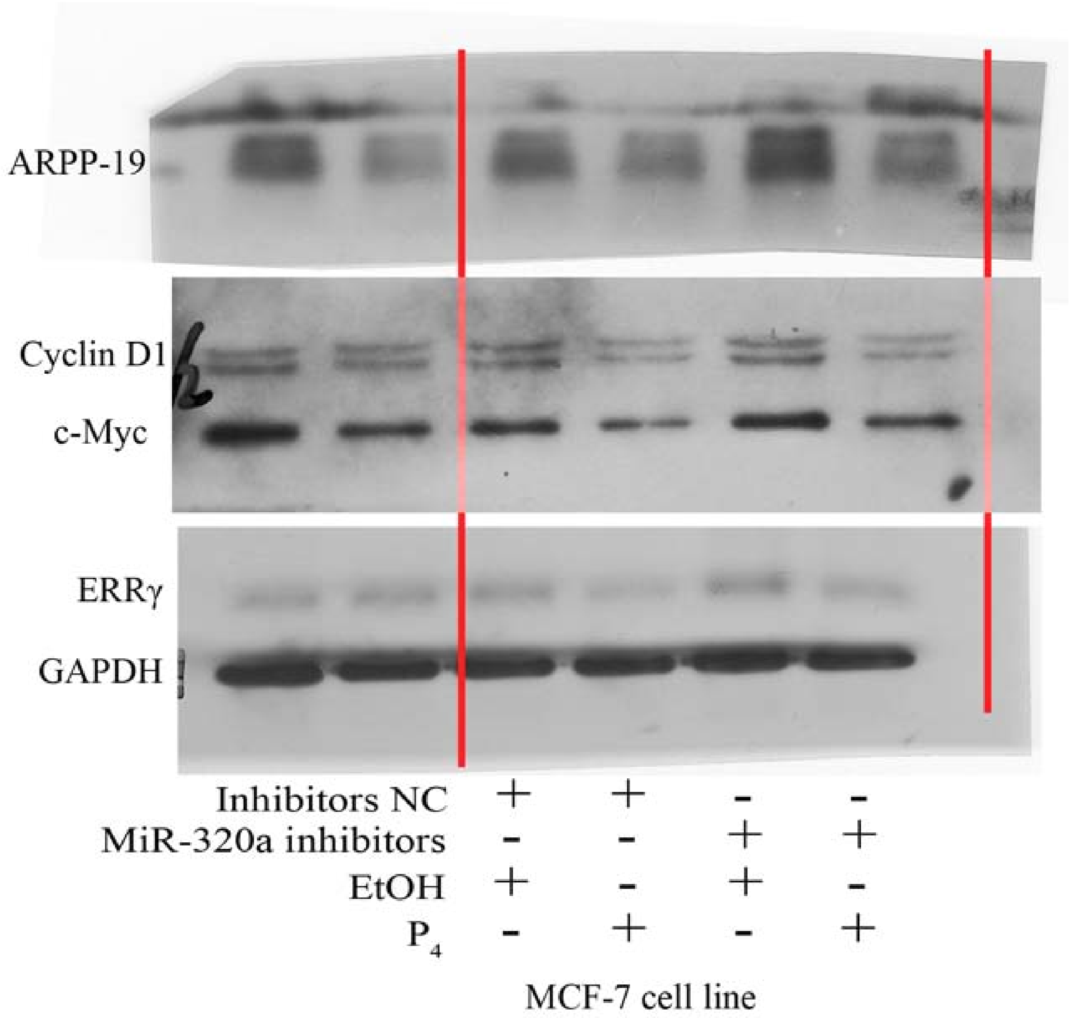

Immunoblot analysis of ARPP-19, ERRγ, c-Myc, and Cyclin D1 levels in MCF-7 cells transfected with miR-320a inhibitors in the presence or absence of P_4 treatment.
